# The dual nature of neuroinflammation in networked brain

**DOI:** 10.3389/fimmu.2025.1659947

**Published:** 2025-08-20

**Authors:** Ludmila Müller, Svetlana Di Benedetto, Viktor Müller

**Affiliations:** Max Planck Institute for Human Development, Center for Lifespan Psychology, Berlin, Germany

**Keywords:** neuroinflammation, brain, neuroimmune interactions, microglia, astrocytes, neurons, CNS, neurological disorders

## Abstract

Neuroinflammation is a dynamic, context-sensitive process that plays essential roles in brain development, maintenance, and response to injury. It reflects a finely balanced neuroimmune state—facilitating repair and adaptation under homeostatic conditions, while also contributing to dysfunction when dysregulated or chronically activated. In this mini-review, we examine the cellular and molecular mechanisms underlying neuroinflammatory responses, focusing on the roles of microglia and astrocytes, their bidirectional communication with neurons, and their interaction with peripheral immune signals. We describe how various stimuli—including aging, protein aggregates, and cellular stress—modulate glial function and shift immune activity toward protective or deleterious outcomes. Special attention is given to endogenous regulatory pathways, including cytokine signaling, receptor-mediated crosstalk, and immunometabolic cues that determine the resolution or persistence of inflammation. We further discuss shared and disease-specific features of neuroinflammation across neurological disorders, offering a systems-level perspective on how immune activity contributes to neural resilience or degeneration. This integrated view aims to inform future studies on neuroimmune dynamics in health and disease.

## 1 Introduction

Neuroinflammation has emerged as a defining feature of numerous neurological and neurodegenerative disorders, yet its role is far from uniform. Rather than a simple marker of pathology, inflammation in the central nervous system (CNS) is a complex, context-dependent process that influences both resilience and degeneration (1–3). In its physiological form, neuroinflammation plays a central role in immune surveillance, synaptic remodeling, and tissue repair. However, upon chronic or uncontrolled activation, it can promote neuronal damage, disrupt homeostasis, and contribute to disease progression (2, 4–6).

The CNS possesses a unique immune environment shaped by tissue-resident glial cells—primarily microglia and astrocytes—and modulated by interactions with neurons, vascular elements, and peripheral immune signals. These cells sense changes in the microenvironment and respond to a wide array of triggers, including infections, trauma, misfolded proteins, and cellular stress. Their responses are guided by tightly regulated signaling pathways that determine whether inflammation resolves, becomes protective, or turns detrimental (3, 7, 8).

A growing body of research has uncovered diverse molecular mechanisms and immunomodulatory cues that influence the course of neuroinflammation (1, 8, 9). While much of the literature has focused on disease-associated inflammation, understanding how immune responses are initiated and regulated under both normal and pathological conditions is critical for unraveling the logic of neuroimmune dynamics.

In this mini-review, we examine the cellular and molecular mechanisms that govern neuroinflammatory responses, highlight key initiating factors, and explore endogenous modulators that shape these responses. By focusing on fundamental processes rather than therapeutic endpoints, we aim to clarify the principles that underlie the dual nature of inflammation in brain health and disease. To understand how neuroinflammation contributes to pathology, it is first crucial to understand its physiological roles. We begin in the next section by examining how immune activity in the healthy brain supports homeostasis, surveillance, and repair.

## 2 Physiological neuroinflammation and immune surveillance

**Figure 1:**
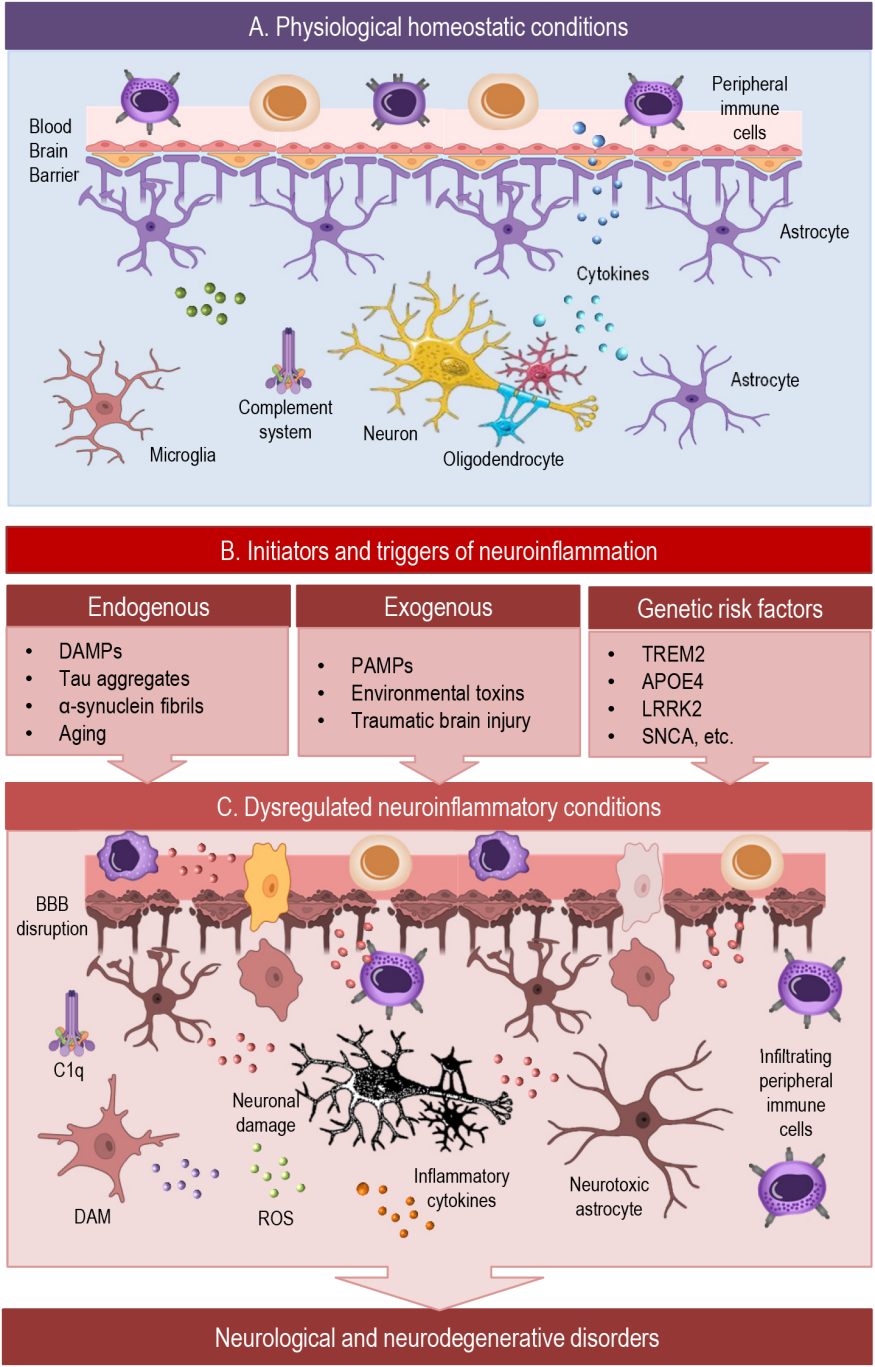
This simplified scheme illustrates dynamic cellular interactions in the brain across physiological and neuroinflammatory conditions. **(A)** Under homeostatic conditions, the CNS maintains balanced immune surveillance through tightly regulated BBB function and glial–neuronal crosstalk. Microglia, astrocytes, and oligodendrocyte-lineage cells support synaptic integrity, modulate neurotransmission, and contribute to developmental pruning and waste clearance. Anti-inflammatory cytokines, along with complement components, sustain a neuroprotective environment and prevent excessive immune activation. **(B)** Diverse initiators —including endogenous damage signals, exogenous pathogens, and genetic risk factors—can shift the brain from a homeostatic to a reactive immune state. These triggers affect glial phenotypes, BBB permeability, and neuroimmune signaling, potentially initiating a cascade toward chronic inflammation. **(C)** In pathological neuroinflammation, sustained activation of glial cells, infiltration of peripheral immune cells, and impaired neuronal feedback create a self-reinforcing loop of inflammatory signaling. Microglia can adopt disease-associated states such as DAM (disease-associated microglia) or LDAM (lipid-droplet-accumulating microglia), which initially aid clearance but may drive chronic inflammation and dysfunction. This dysregulation contributes to BBB disruption, synaptic impairment, and progressive neuronal damage, promoting the development of neurological and neurodegenerative disorders.BBB, blood-brain barrier; C1q, complement component; DAM, disease associated microglia; ROS, reactive oxygen species.

Neuroinflammation is often associated with pathology, but low-level immune activity is a normal and essential feature of CNS physiology. To recognize the full spectrum of neuroinflammatory responses, it is essential to first consider their roles under physiological conditions (**Figure 1A**). Even in the absence of injury or disease, the central nervous system relies on tightly regulated immune activity to maintain homeostasis (1, 8). Glial cells continuously monitor the neural environment, modulate synaptic function, and engage in crosstalk with neurons and the vasculature. These baseline immune functions are not only non-disruptive but are integral to normal brain development, plasticity, and repair (9–12). Exploring this foundational role of neuroinflammation reveals its adaptive potential—and sets the stage for understanding how these same processes may become maladaptive in pathology.

In the healthy brain, neuroinflammatory signaling contributes to homeostatic surveillance, synaptic regulation, and tissue maintenance (8). Though often described as immunologically privileged, the CNS is in continuous biochemical communication with the periphery. The blood–brain barrier (BBB) regulates this exchange, maintaining immune selectivity through tightly controlled permeability. Under physiological conditions, peripheral immune cells are largely excluded from the parenchyma, but antigen-presenting signals and cytokine gradients cross the BBB, informing systemic immune tone (13–15). Meningeal lymphatics and perivascular spaces further contribute to immune dialogue between brain and body (16–18).

Glial cells —primarily microglia, astrocytes, and oligodendrocyte-lineage cells—play central roles in this baseline immune state, dynamically sensing the neural environment and communicating with both local and peripheral immune components. In steady-state conditions, these cell types contribute to a finely tuned immunological environment that supports neuronal communication and structural integrity (8).

Microglia, the resident immune cells of the CNS, continuously survey their surroundings through highly motile processes (19, 20). Even in the absence of injury, they engage in synaptic pruning, phagocytosis of cellular debris, and secretion of trophic factors that support neuronal function (19, 20). Their resting phenotype is defined by a unique transcriptional and metabolic profile that maintains sensitivity to subtle environmental changes (1, 21).

Astrocytes also show active participation in immune surveillance, regulating extracellular ion balance, neurotransmitter clearance, and synapse maturation (10). In addition, astrocytes support BBB function, fine-tune synaptic signaling, and facilitate the removal of extracellular waste and infectious agents. Upon activation, astrocytes secrete a diverse array of pro- and anti-inflammatory mediators—such as cytokines, chemokines, growth factors, and reactive oxygen species—that modulate the function of nearby neurons, microglia, and endothelial cells (10, 22–25).

Beyond serving as progenitors for myelinating oligodendrocytes, oligodendrocyte precursor cells (OPCs) are increasingly recognized as active players in CNS immune surveillance. Identified by markers such as NG2 and PDGFRα, OPCs are widely distributed in the adult brain and remain highly responsive to changes in their environment (26, 27). Even under steady-state conditions, they express pattern recognition receptors and can detect inflammatory cues, positioning them as sensitive sensors of tissue stress. In addition to contributing to remyelination, OPCs influence extracellular matrix composition and synaptic activity, highlighting their broader role in maintaining homeostatic balance within the glial network. OPCs also engage in crosstalk with astrocytes and microglia, modulating immune tone through cytokine signaling and extracellular vesicles (26–28).

Cytokines are small signaling proteins that regulate neuronal function, synaptic plasticity, immune responses, and tissue repair, playing a dual role in both brain homeostasis and neuroinflammation (29, 30). The cytokine network, present throughout the brain and body, is tightly regulated across the lifespan. Through complex cascades, cytokines act synergistically or antagonistically to mediate cell–cell communication and translate environmental signals into cellular responses (2, 31). Homeostatic levels of cytokines such as interleukin-10 (IL-10), transforming growth factor-beta (TGF-β), and fractalkine (CX3CL1) help sustain a non-inflammatory, neuroprotective milieu (32, 33).

Complement components, traditionally associated with immune defense, also play developmental roles in synaptic tagging and elimination, particularly during early brain maturation (34). The balance of cytokine and complement signaling ensures immune readiness while preventing excessive activation that could disturb neural function (34, 35).

A variety of cell-intrinsic and intercellular mechanisms maintain the immune balance in the CNS. Neurons express ligands such as CD200 and CX3CL1, which signal to glial cells via their respective receptors (CD200R and CX3CR1) to suppress pro-inflammatory responses. Microglia and astrocytes also produce immunoregulatory molecules including TGF-β and IL-10, reinforcing a homeostatic loop. These baseline signals enable rapid but restrained responses to perturbations, preventing unnecessary inflammation while preserving readiness to respond to damage or infection (8, 10, 33, 36).

Together, these physiological mechanisms ensure that immune surveillance in the CNS remains balanced, responsive, and non-disruptive to neural function. However, when homeostatic control is challenged, a wide range of endogenous and exogenous triggers can shift neuroinflammation toward a reactive or pathological state—a transition we explore in the following section.

## 3 Initiators and triggers of neuroinflammatory responses

Neuroinflammation is a complex biological process initiated by diverse triggers that activate resident immune cells within the CNS, primarily microglia and astrocytes (4, 37). These triggers can be broadly categorized into endogenous and exogenous factors, each contributing to the initiation, amplification, and chronicity of neuroinflammatory responses (**Figure 1B**).

Endogenous triggers originate internally, reflecting cellular distress or pathological changes within CNS tissue. A central group of molecules involved are damage-associated molecular patterns (DAMPs), including ATP, high-mobility group box 1 (HMGB1), heat-shock proteins, and mitochondrial DNA (38, 39). These DAMPs bind to pattern recognition receptors (PRRs) such as Toll-like receptors (TLRs, e.g., TLR4) and nucleotide-binding oligomerization domain-like receptors (NLRs, e.g., NLRP3 inflammasome) expressed on microglia and astrocytes. This interaction triggers downstream signaling cascades and inflammasome activation, resulting in secretion of pro-inflammatory cytokines such as IL-1β and TNF-α (40–42).

Another critical source of endogenous neuroinflammatory triggers is the accumulation of protein aggregates that are hallmarks of neurodegenerative diseases. Misfolded amyloid-β (Aβ) peptides activate microglia via receptors including TLRs and scavenger receptors, promoting a sustained inflammatory state (42, 43). Tau aggregates stimulate inflammasome pathways and contribute to microglial priming (44). Similarly, α-synuclein fibrils implicated in Parkinson’s disease bind to TLR2 and induce pro-inflammatory cytokine release (45).

Aging itself is a major endogenous factor predisposing the CNS to heightened inflammation (2, 46). Aging leads to a chronic low-grade pro-inflammatory state termed inflammaging, characterized by increased basal cytokine levels, microglial priming, and impaired resolution mechanisms (47). Metabolic stress, such as mitochondrial dysfunction, elevates production of reactive oxygen species (ROS) and promotes activation of the NLRP3 inflammasome, exacerbating neuroinflammation (48).

Remarkably, beyond classical immune cells, a diverse array of non-immune cell types—including mesenchymal stromal/stem cells, fibroblasts, endothelial cells, osteoblasts, neurons, and Schwann cells—also exhibit essential immune-regulatory functions that may become dysregulated with aging. These include the secretion of cytokines, chemokines, and growth factors, as well as roles in promoting inflammation, presenting antigens, exerting immunosuppressive effects, and mounting antimicrobial responses, particularly under conditions of infection or inflammation (10, 49). With aging, these immunological functions may become impaired, leading to dysregulated cellular activity and contributing to the pathogenesis of age-related diseases and neurodegeneration.

Exogenous triggers arise from environmental or pathogenic insults. Pathogen-associated molecular patterns (PAMPs) derived from bacterial lipopolysaccharides (LPS), viral RNA, or fungal components activate TLRs and other PRRs on microglia and astrocytes. This activation provokes innate immune responses, including the release of cytokines and chemokines, as well as the recruitment of peripheral immune cells (50, 51). Environmental toxins, such as pesticides, heavy metals, and air pollutants, induce oxidative stress and mitochondrial damage, indirectly activating glial inflammatory pathways (52, 53). Traumatic brain injury (TBI) causes mechanical damage that leads to the release of DAMPs and BBB disruption, amplifying CNS immune activation and chronic neuroinflammation (54).

Genetic variants critically shape neuroinflammatory sensitivity and the efficacy of immune responses within the central nervous system. They influence how resident immune cells, such as microglia and astrocytes, detect and respond to endogenous and exogenous triggers, thereby modulating both protective and pathological inflammation. By altering receptor function, signaling pathways, and cellular metabolism, specific gene variants can either amplify or dampen neuroinflammatory cascades, ultimately impacting the onset, progression, and severity of various neurological disorders. For instance, the microglial receptor TREM2 regulates phagocytosis and inflammatory modulation. Loss-of-function mutations reduce clearance of apoptotic neurons and protein aggregates, leading to chronic inflammation (55). TREM2 signaling promotes a neuroprotective microglial phenotype through DAP12-mediated pathways, modulating PI3K-Akt and suppressing excessive nuclear factor kappa B (NF-κB) activation (56).

Another example is the APOE4 allele, which influences lipid metabolism and neuroinflammation. APOE4 carriers exhibit disrupted BBB integrity, increased microglial activation, and impaired clearance of Aβ (57, 58). APOE4 modulates TLR signaling and inflammasome activation, contributing to a pro-inflammatory milieu (59). These genetic predispositions not only amplify neuroinflammatory triggers but also interfere with resolution and repair, shaping disease susceptibility and progression in disorders such as Alzheimer’s disease (AD) and Parkinson’s disease (PD).

In summary, a broad spectrum of endogenous and exogenous stimuli—including DAMPs, PAMPs, protein aggregates, metabolic stress, and environmental insults—can initiate and shape neuroinflammatory responses in the central nervous system. The magnitude and character of these responses are further shaped by genetic risk factors such as TREM2 and APOE4, which modulate immune sensitivity and glial reactivity. Together, these elements form the initiating framework of neuroinflammation, determining how the CNS perceives and reacts to various perturbations. To understand how these initial triggers are translated into coordinated cellular behaviors and signaling pathways, the following section explores the cellular and molecular mechanisms governing neuroinflammation.

## 4 Cellular and molecular mechanisms governing neuroinflammation

Once initiated by endogenous or exogenous stimuli, neuroinflammatory responses (**Figure 1C**) are mediated by CNS-resident cells, primarily microglia and astrocytes, which detect danger signals and activate context-specific molecular programs. These involve tightly regulated signaling pathways—such as NF-κB, Janus kinase/signal transducer and activator of transcription (JAK/STAT), mitogen-activated protein kinase (MAPKs), and inflammasomes—that govern cytokine and chemokine production, oxidative stress responses, and intercellular communication (60, 61). Through dynamic interactions with neurons, endothelial cells, and, when relevant, infiltrating immune cells, glial responses shape the local inflammatory milieu (37). The following subsections detail the cellular roles of microglia and astrocytes and the intracellular mechanisms driving their activation.

### 4.1 Microglial and astrocytic phenotypic changes

Microglia and astrocytes exhibit remarkable phenotypic plasticity in response to neuroinflammatory triggers (**Figure 1C**), adapting their functional states along dynamic spectrums rather than fixed binary polarizations (1, 62–64). Historically, microglial activation has been described by the M1/M2 classification, where M1 microglia adopt a pro-inflammatory profile characterized by production of cytokines such as TNF-α, IL-1β, and reactive oxygen species, while M2 microglia promote tissue repair and anti-inflammatory responses.

However, this binary framework oversimplifies the complexity of microglial responses. Recent single-cell and spatial transcriptomic studies have uncovered a spectrum of microglial phenotypes shaped by stimulus type, age, spatial niche, and disease progression. For instance, disease-associated microglia (DAM) and lipid-droplet-accumulating microglia (LDAM) are distinct microglial states linked to neurodegeneration and aging, marked by altered metabolism, impaired phagocytosis, and heightened inflammatory activity (19, 64–70). These dynamic microglial states have pivotal implications for therapy: interventions should aim to selectively modulate specific phenotypes or signaling pathways—depending on disease stage, spatial context, and the balance between protective and deleterious functions (71, 72).

Similarly, astrocytes undergo reactive changes that range from neuroprotective (A2) to neurotoxic (A1) phenotypes (1, 73–75). Neurotoxic astrocytes, induced by microglia-derived factors such as IL-1α, TNF-α, and C1q, lose normal supportive functions and release neurotoxic mediators contributing to neuronal injury and degeneration (**Figure 1C**). In contrast, A2 astrocytes are associated with neuroprotection and repair, producing growth factors and anti-inflammatory molecules (**Figure 1A**). Like microglia, astrocyte activation is highly context-dependent and involves complex transcriptional and epigenetic regulation (76, 77). However, similar to the M1/M2 paradigm in microglia, these classifications oversimplify the diverse and dynamic range of astrocytic responses in the inflamed nervous system (1, 78, 79).

Importantly, microglial and astrocytic phenotypes are not isolated states but reflect continuous adaptations within an interconnected cellular network. Their activation profiles shape the local cytokine milieu, influence blood-brain barrier integrity, and regulate recruitment of peripheral immune cells, ultimately determining the progression or resolution of neuroinflammation.

### 4.2 Signaling pathways

The activation and functional responses of microglia and astrocytes during neuroinflammation are governed by multiple intracellular signaling cascades that integrate external stimuli into specific transcriptional programs. Among these, the NF-κB pathway is a central regulator of pro-inflammatory gene expression, controlling the release of cytokines, chemokines, and adhesion molecules (80). Activation of NF-κB typically occurs downstream of pattern recognition receptors such as TLRs and leads to rapid induction of inflammatory mediators.

Additionally, several intracellular signaling pathways play pivotal roles in sensing cellular stress, regulating immune responses, and mediating communication between neurons, glia, and immune cells. Among the most prominent are the NLRP3 inflammasome, the JAK/STAT pathway, the MAPK cascades, and the cGAS–STING pathway—each contributing to the detection of danger signals and the orchestration of downstream inflammatory processes within the central nervous system.

The NLRP3 inflammasome is a critical cytosolic multiprotein complex that senses cellular stress and danger signals, triggering caspase-1 activation and subsequent maturation of IL-1β and IL-18. This pathway contributes to the amplification of neuroinflammation and is implicated in numerous neurodegenerative diseases (43).

The JAK/STAT pathway mediates responses to a variety of cytokines and growth factors. In particular, STAT3 activation in astrocytes is associated with both protective and detrimental effects depending on the inflammatory context, influencing astrogliosis and scar formation (60, 81, 82). This pathway plays a key role in modulating neuroinflammatory processes by shaping glial reactivity, regulating immune cell recruitment, and sustaining chronic inflammation when dysregulated.

The MAPK cascades, including extracellular signal-regulated kinase (ERK), c-Jun N-terminal kinase (JNK), and p38, regulate diverse aspects of glial activation, from cytokine production to cell survival and apoptosis. These pathways often cross-talk with NF-κB and JAK/STAT signaling to fine-tune inflammatory responses (83). Their sustained activation has been linked to chronic neuroinflammation and progressive neuronal damage in various CNS disorders.

Finally, the cyclic GMP-AMP synthase–stimulator of interferon genes (cGAS–STING) pathway detects cytosolic DNA from pathogens or damaged cells, leading to type I interferon production and an antiviral state. Emerging evidence implicates cGAS–STING signaling in sterile neuroinflammation and neurodegeneration, highlighting its importance in CNS immune surveillance (84, 85).

Together, these signaling pathways coordinate glial responses to injury and infection, balancing host defense with tissue preservation. Dysregulation at any level can contribute to chronic inflammation and neuronal damage.

### 4.3 Crosstalk and feedback regulation

Microglia and astrocytes engage in extensive bidirectional communication that shapes the magnitude, duration, and outcome of neuroinflammatory responses (8, 37). Microglia-derived cytokines such as IL-1α, TNF-α, and complement component (C1q) can induce reactive astrocyte phenotypes, notably the neurotoxic A1 state (86, 87). Conversely, astrocytes modulate microglial activation by releasing anti-inflammatory factors like TGF-β, IL-10, and ATP-degrading enzymes that dampen purinergic signaling. These reciprocal influences are dynamically regulated and context-dependent, enabling either amplification or resolution of inflammation (88).

Feedback mechanisms also arise through autocrine and paracrine signaling. For instance, sustained activation of NF-κB or STAT3 can reinforce inflammatory gene expression in both glial types, while negative regulators such as suppressor of cytokine signaling (SOCS) proteins, A20, and microRNAs act to constrain excessive responses (89). Additionally, metabolic cues—such as shifts in glycolysis or oxidative phosphorylation—affect glial activation states and modulate the inflammatory tone via immunometabolic pathways (90).

The integrity of these crosstalk mechanisms is essential for maintaining CNS homeostasis. Disruption of feedback regulation—due to aging, chronic stress, or genetic susceptibility—can lead to persistent glial activation, increased cytokine load, and secondary damage to neurons and oligodendrocytes. Understanding these interactions provides key insights into how glial networks adapt to inflammatory challenges and why these processes may fail in disease.

### 4.4 Immune–neural communication loops

Neuroinflammatory responses are tightly shaped by the bidirectional interactions between neurons and glial cells. Neurons are not passive bystanders in neuroinflammation but actively participate in shaping glial responses through finely tuned immune–neural communication loops. Under homeostatic conditions, neurons express “off” signals—such as CD200, fractalkine, and TGF-β—that engage receptors on microglia and astrocytes to maintain them in a surveillant, non-inflammatory state (91). During stress or injury, neuronal signaling patterns change significantly, with altered expression of danger signals, DAMPs, and altered neurotransmitter release that can drive glial activation (8, 37, 91, 92).

Conversely, reactive glia profoundly influence neuronal function (**Figure 1C**). Microglia and astrocytes release cytokines (e.g., IL-1β, TNF-α), reactive oxygen species, and glutamate, which can impair synaptic transmission, disrupt neuronal excitability, and induce cell death. Astrocytic loss of homeostatic functions—such as glutamate uptake and ion buffering—further exacerbates neuronal stress. Importantly, chronic or unresolved neuroinflammation can alter synaptic pruning, plasticity, and long-term neuronal viability, contributing to cognitive and behavioral dysfunction (8, 37, 93, 94).

These bidirectional loops are tightly regulated under physiological conditions but become dysregulated in neurodegenerative and neuropsychiatric disorders. The failure of neurons to restrain glial activation, or the persistence of glia-derived neurotoxic signals, establishes a self-reinforcing inflammatory circuit that promotes disease progression. Deciphering the molecular mediators and timing of these immune–neural interactions is crucial for identifying therapeutic strategies that restore balance and protect neural function in the inflamed CNS (8, 37).

Thus, neuroinflammation emerges from a complex and dynamic interplay between glial cells, signaling pathways, and neuron-glia communication. Microglia and astrocytes adopt diverse phenotypes in response to environmental cues, executing both protective and detrimental functions. These responses are orchestrated through interconnected intracellular pathways and tightly regulated by reciprocal glial crosstalk and neuronal input. Disruption of these regulatory networks—through chronic stimulation, aging, or genetic vulnerability—can shift glial responses toward sustained inflammation and neurotoxicity. Understanding the mechanisms that govern this cellular network is key to identifying points of intervention for modulating neuroinflammation in neurological diseases.

## 5 Neuroinflammation in disease contexts: mechanistic insights

While the cellular and molecular mechanisms of neuroinflammation share common elements across conditions, their specific manifestation varies significantly depending on the disease context. In each disorder, distinct triggers, temporal dynamics, and cellular environments shape the nature and outcome of the inflammatory response. Whether inflammation serves a reparative, neutral, or detrimental role is determined by the interplay of acute versus chronic activation, the affected brain region, and underlying genetic and systemic influences.

Neuroinflammation is increasingly recognized as a key contributor to the pathophysiology of various neuropsychiatric disorders, including major depressive disorder, schizophrenia, and bipolar disorder (95–97). In these conditions, altered glial activation, elevated levels of pro-inflammatory cytokines (e.g., IL-6, TNF-α), and disrupted BBB integrity have been observed, even in the absence of overt neurodegeneration (98). Microglia and astrocytes exhibit region-specific changes in reactivity, particularly within the prefrontal cortex, hippocampus, and amygdala—areas critical for mood and cognition. Dysregulated immune-to-brain signaling, often involving peripheral immune activation or stress-induced HPA axis dysfunction, may prime glial cells toward a pro-inflammatory state, contributing to synaptic alterations and behavioral symptoms. Moreover, emerging evidence links inflammatory profiles with treatment resistance in depression, and clinical trials are underway to evaluate anti-inflammatory agents as adjunctive therapies (99, 100). These findings underscore the importance of considering glial–immune dynamics not only in neurodegeneration but also in the broader landscape of brain disorders affecting cognition, emotion, and behavior.

**Figure 2:**
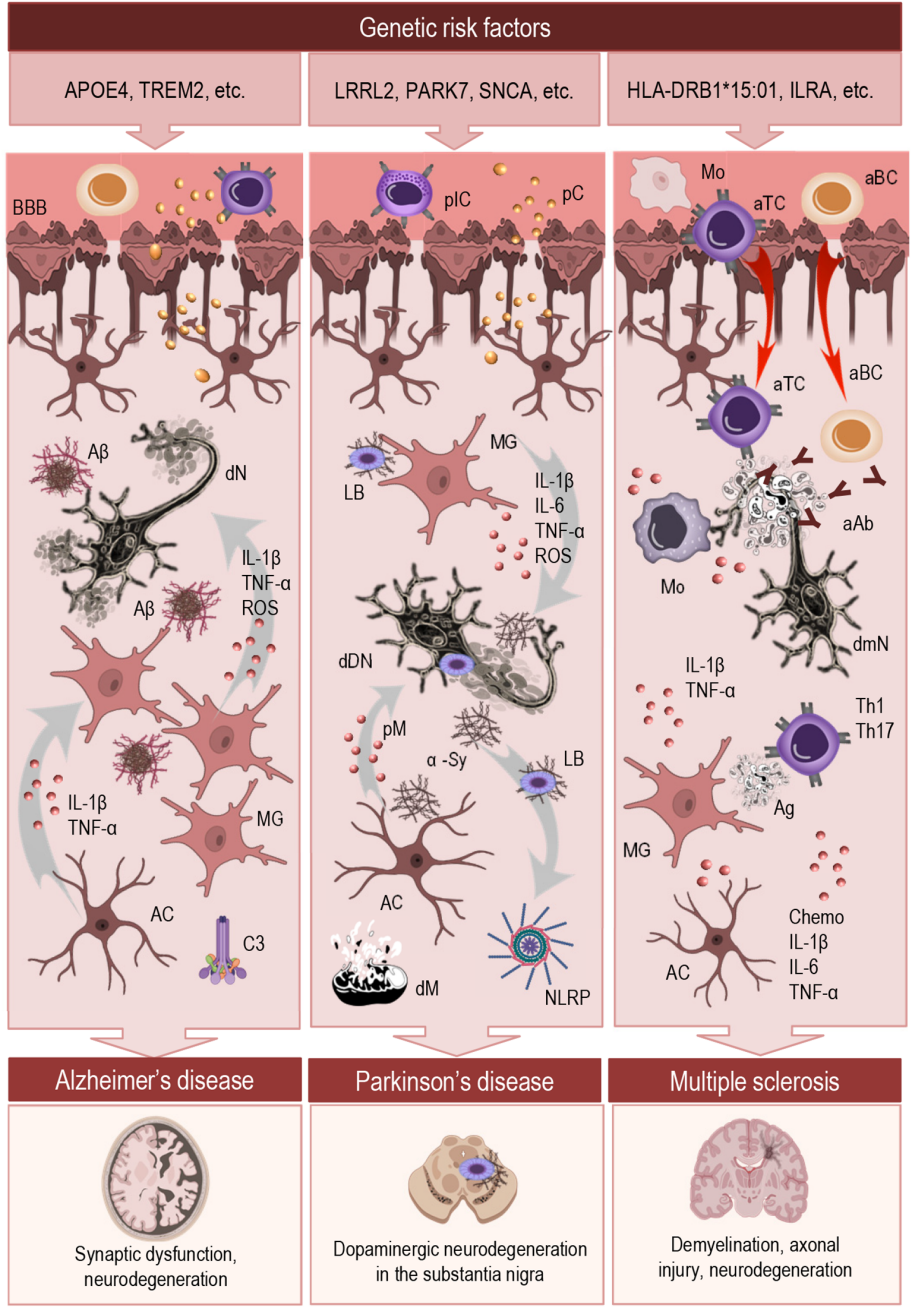
Mechanistic overview of neuroinflammation across major neurodegenerative diseases. This schematic illustrates the initiation and regulation of neuroinflammatory responses in AD, PD, and MS. The top panel highlights key genetic risk factors associated with each condition. The central section outlines shared and disease-specific inflammatory mechanisms, including innate immune responses to protein aggregates (AD, PD), autoimmune infiltration (MS), and chronic activation of microglia and astrocytes. These immune processes converge on a maladaptive inflammatory state that disrupts CNS homeostasis and promotes neuronal dysfunction. Despite their distinct etiologies, all three disorders involve persistent glial dysregulation, impaired resolution, and sustained neuroinflammation. The bottom panel depicts characteristic pathological outcomes associated with each disease. MG, microglia; AC, astrocyte; BBB, blood-brane-barrier; IL, interleukin; TNF, tumor necrosis factor; ROS, reactive oxygen species; pIC, peripheral immune cells; pC, peripheral cytokines; Mo, monocytes; aTC, autoreactive T cells; aBC, autoreactive B cells; aAb, auto-antibodies; Aβ, amyloid-β; dN, degenerating neuron; C3, complement component; LB, Lewis body; α-Sy, α-synuclein; dDN, degenerating dopaminergic neuron; pM, proinflammatory mediators; NLRP, inflammasome; dM, dysfunctional mitochondria; dmN, degenerating demyelinated neuron; Chemo, chemokines.

The following section highlights shared and divergent neuroinflammatory mechanisms in selected neurological diseases, such as AD, PD, and multiple sclerosis (MS), with a focus on how context influences glial reactivity, immune signaling, and the transition from protective responses to chronic neurotoxicity (**Figure 2**).

### 5.1 Alzheimer’s disease

In AD (**Figure 2**, central section, left) neuroinflammation is a prominent and early feature that contributes to disease onset and progression. Activated microglia cluster around amyloid-β (Aβ) plaques and release pro-inflammatory mediators such as IL-1β, TNF-α, and reactive oxygen species, contributing to synaptic dysfunction and neuronal loss. While initially recruited to clear Aβ aggregates, microglia in the AD brain often become chronically activated and adopt a dysfunctional phenotype that is inefficient in phagocytosis but sustained in cytokine release (101, 102). This shift contributes to a toxic feedback loop, wherein persistent inflammation exacerbates Aβ pathology and tau hyperphosphorylation.

Astrocytes also exhibit reactive changes in AD, displaying both hypertrophy and altered expression of inflammatory genes. Reactive astrocytes can propagate inflammation through complement activation (e.g., C3), impaired glutamate clearance, and altered metabolic support to neurons. The interplay between microglia and astrocytes, particularly the induction of neurotoxic A1 astrocytes by microglia-derived signals (e.g., IL-1α, TNF-α), is increasingly recognized as a key contributor to neuronal vulnerability in AD (103).

Genetic risk factors (**Figure 2**, top panel, left) further sensitize the neuroinflammatory response. APOE4, the strongest genetic risk factor for late-onset AD, is associated with impaired Aβ clearance and increased pro-inflammatory glial activity (104). Similarly, TREM2 variants, which affect microglial survival and lipid sensing, modulate microglial responses to Aβ and influence plaque compaction and surrounding inflammation (105, 106). These findings highlight that inflammation in AD is not merely a secondary consequence but an active driver of neurodegeneration, shaped by both intrinsic genetic programs and ongoing pathological stimuli.

### 5.2 Parkinson’s disease

Neuroinflammation is increasingly recognized as a critical component of PD pathogenesis (**Figure 2**, central section, middle), contributing to the progressive loss of dopaminergic neurons in the substantia nigra (107, 108). Microglia in PD brains display a persistently activated phenotype, characterized by upregulation of MHC class II, elevated pro-inflammatory cytokine release (e.g., TNF-α, IL-6, IL-1β), and increased production of reactive oxygen and nitrogen species. This sustained pro-inflammatory state not only damages neurons directly but also promotes α-synuclein aggregation and impairs its clearance, perpetuating a harmful feed-forward loop (107, 109, 110).

Misfolded and aggregated α-synuclein acts as a potent DAMP, triggering innate immune receptors such as TLRs and the NLRP3 inflammasome. These pathways initiate and amplify microglial activation, leading to caspase-1–mediated IL-1β release and pyroptotic responses. Astrocytes also respond to α-synuclein and contribute to neuroinflammation by producing pro-inflammatory mediators, exhibiting impaired neurotrophic support, and engaging in dysfunctional glutamate homeostasis (109, 111).

Genetic mutations associated with familial PD (**Figure 2**, top panel, middle), such as those in leucine-rich repeat kinase 2 (LRRK2) and Parkinsonism associated deglycase (PARK7, also known as DJ-1), as well as SNCA (α-synuclein), influence inflammatory susceptibility by altering mitochondrial function, oxidative stress responses, and autophagy. Notably, LRRK2 is highly expressed in immune cells and regulates inflammatory signaling, linking genetic vulnerability to dysregulated immune responses in PD (112–114).

Together, these mechanisms underscore the central role of innate immune dysfunction in PD. Unlike in AD, where inflammation may precede overt neurodegeneration, in PD it appears to act in concert with proteinopathy and mitochondrial dysfunction, forming a triad of pathological drivers.

### 5.3 Multiple sclerosis

Multiple sclerosis is a chronic autoimmune disorder of the central nervous system in which neuroinflammation is a central pathogenic mechanism driving demyelination, axonal injury, and neurodegeneration (115). Unlike neurodegenerative diseases where inflammation arises primarily from intrinsic CNS signals, MS is characterized by the infiltration of peripheral immune cells—including autoreactive T cells, B cells, and monocytes—across a compromised BBB (**Figure 2**, central section, right). These cells interact with resident microglia and astrocytes, amplifying local inflammation and tissue damage (116).

Microglia in MS lesions are highly reactive and contribute to both early demyelination and chronic lesion expansion. They express pro-inflammatory cytokines (e.g., IL-1β, TNF-α), present antigens, and produce reactive oxygen species and nitric oxide, all of which exacerbate oligodendrocyte injury. In chronic active lesions, microglia form a rim around slowly expanding plaques, maintaining a smoldering inflammatory state associated with disease progression (115, 117).

Astrocytes contribute to both the propagation and modulation of neuroinflammation in MS. They upregulate chemokines (e.g., CCL2, CCL5, CXCL10) and adhesion molecules (e.g., VCAM-1, ICAM-1) promoting immune cell infiltration across the blood–brain barrier. Astrocyte-derived cytokines, including IL-1β, IL-6, TNF-α, and TGF-β, influence T-cell polarization and interactions with microglia, further shaping the inflammatory milieu. However, astrocytes also play protective roles by promoting BBB repair, producing anti-inflammatory mediators, and supporting remyelination, illustrating their dual role in MS pathophysiology (115, 118).

In multiple sclerosis, peripheral immune dysregulation—characterized by the activation of autoreactive T and B cells—is recognized as a primary driver of CNS pathology. These cells infiltrate a compromised BBB and initiate CNS inflammation, where subsequent interactions with resident microglia and astrocytes amplify local neuroimmune responses, forming a pathogenic feedback loop that sustains demyelination and neurodegeneration (119).

Building on this, peripheral immune activity remains functionally interconnected with immune responses within the CNS. Pro-inflammatory Th1 and Th17 cells contribute to tissue damage, while dysfunction of regulatory T cells impairs immune resolution and promotes disease persistence (120, 121). B cells also play an increasingly important role in MS through antibody production and cytokine secretion, with therapies targeting B cells (e.g., anti-CD20 monoclonals) showing clinical efficacy (122, 123).

The strongest genetic risk factor for MS is the HLA-DRB1*15:01 allele, located in the MHC class II region, which increases disease risk by facilitating autoreactive CD4^+^ T cell activation against CNS antigens (124). Among non-HLA genes, IL2RA, encoding the interleukin-2 receptor alpha chain (CD25), has been linked to impaired regulatory T cell function, contributing to immune dysregulation and loss of tolerance (125, 126). These variants underscore the central role of adaptive immunity in MS pathogenesis (**Figure 2**, top panel, right).

Thus, MS exemplifies a context in which neuroinflammation arises from both peripheral and central immune mechanisms. The spatial and temporal dynamics of glial activation, immune cell infiltration, and lesion evolution define the clinical heterogeneity of MS and provide therapeutic entry points for immunomodulation.

Together, AD, PD, and MS illustrate how neuroinflammation contributes to CNS pathology (**Figure 2**, bottom panel) through distinct yet overlapping mechanisms. From innate immune responses to protein aggregates, to autoimmune infiltration and chronic glial activation, these disorders underscore the central role of context-specific inflammatory networks in shaping disease trajectories. Despite differences in etiology, all involve dysregulated microglial and astrocytic activity, persistent signaling imbalances, and compromised resolution, which ultimately fuel neurodegeneration. These insights highlight not only the complexity of neuroinflammatory processes but also the pressing need for refined tools and conceptual frameworks to study them.

## 6 Conclusions and future directions

Neuroinflammation emerges as a central and dynamic process across a range of neurological disorders, shaped by disease-specific triggers, glial phenotypic plasticity, and complicate signaling networks. This mini-review highlights that while the cellular and molecular mechanisms vary across conditions such as AD, PD, and MS, they converge on common principles: chronic glial activation, impaired resolution, and maladaptive immune-neural interactions that perpetuate neurodegeneration. A nuanced understanding of these processes is essential to advance the field beyond oversimplified models and toward context-aware, mechanistic insight.

Looking ahead, a shift toward system-level frameworks is essential to capture the full complexity of neuroimmune interactions. Rather than focusing solely on individual cell types or signaling pathways, future research must model inflammation as an emergent property of networked communication—shaped by feedback, crosstalk, and spatial context within the CNS. This perspective is particularly valuable for understanding how inflammation evolves over time and how its resolution or persistence impacts disease progression.

A deeper understanding of neuroimmune interactions requires moving beyond linear cause-effect models toward systems that reflect the true complexity of brain function. Multilayer network models offer a powerful framework to capture the dynamic interplay among diverse cellular populations, signaling pathways, and environmental influences. These models allow for the integration of molecular, cellular, and circuit-level data across spatial and temporal scales, revealing emergent properties that are not apparent in isolated datasets. By mapping how immune signals propagate through neural circuits and how glial responses influence synaptic and systemic functions, multilayer approaches can uncover key nodes of vulnerability or resilience. This systems perspective is crucial for identifying intervention points and for designing strategies that modulate neuroinflammation with greater precision and fewer unintended consequences.

Equally vital is the pressing need to refine our understanding of glial diversity through high-resolution phenotyping. Traditional classifications, such as the M1/M2 and A1/A2 dichotomies, offer only a limited view of the dynamic and context-dependent states glial cells assume *in vivo*. Advances in single-cell transcriptomics, spatial profiling, and high-resolution imaging now open the door to a more nuanced and integrative characterization of glial function across health, aging, and disease.

Advancing our understanding of neuroinflammation demands integrative, high-resolution, and systems-oriented approaches that can capture its complexity across molecular, cellular, and circuit levels. By implementation multilayered network models, we will be more able to unravel the dichotomous function of neuroinflammation, clarify the delicate balance between protective and pathological immune responses in the brain, and identify meaningful points of intervention for future translational research.

Neuroinflammation represents a dynamic and multifaceted process within the highly interconnected cellular landscape of the brain. Rather than acting as a singular harmful force, inflammatory responses serve both protective and pathogenic roles, depending on their intensity, duration, and cellular context. Acute, well-regulated neuroinflammation is essential for host defense, tissue repair, and the restoration of homeostasis. In contrast, chronic or dysregulated inflammatory signaling—shaped by genetic predispositions, aging, and environmental insults—can lead to glial dysfunction, synaptic alterations, and progressive neurodegeneration. Understanding this dualistic nature is crucial for decoding the complex interplay between immune signaling and neural function in health and disease.

## References

[1] Nosi D Lana D Giovannini MG Delfino G Zecchi-Orlandini S. Neuroinflammation: Integrated Nervous Tissue Response through Intercellular Interactions at the “Whole System” Scale. Cells. (2021) 10. doi: 10.3390/cells10051195, PMID: 34068375 PMC8153304

[2] Di Benedetto S Müller L Wenger E Duzel S Pawelec G. Contribution of neuroinflammation and immunity to brain aging and the mitigating effects of physical and cognitive interventions. Neurosci Biobehav Rev. (2017) 75:114–28. doi: 10.1016/j.neubiorev.2017.01.044, PMID: 28161508

[3] Chitnis T Weiner HL. CNS inflammation and neurodegeneration. J Clin Invest. (2017) 127:3577–87. doi: 10.1172/JCI90609, PMID: 28872464 PMC5617655

[4] DiSabato DJ Quan N Godbout JP. Neuroinflammation: the devil is in the details. J Neurochem. (2016) 139 Suppl 2:136–53. doi: 10.1111/jnc.13607, PMID: 26990767 PMC5025335

[5] Sierra A Abiega O Shahraz A Neumann H. Janus-faced microglia: beneficial and detrimental consequences of microglial phagocytosis. Front Cell Neurosci. (2013) 7:6. doi: 10.3389/fncel.2013.00006, PMID: 23386811 PMC3558702

[6] Rigillo G Alboni S. Exploring the frontiers of neuroinflammation: new horizons in research and treatment. Curr Issues Mol Biol. (2024) 46:11665–7. doi: 10.3390/cimb46100692, PMID: 39451572 PMC11506371

[7] Li H Ghorbani S Ling CC Yong VW Xue M. The extracellular matrix as modifier of neuroinflammation and recovery in ischemic stroke and intracerebral hemorrhage. Neurobiol Dis. (2023) 186:106282. doi: 10.1016/j.nbd.2023.106282, PMID: 37683956

[8] Müller L Di Benedetto S Müller V. From homeostasis to neuroinflammation: insights into cellular and molecular interactions and network dynamics. Cells. (2025) 14. doi: 10.3390/cells14010054, PMID: 39791755 PMC11720143

[9] Silbereis JC Pochareddy S Zhu Y Li M Sestan N. The cellular and molecular landscapes of the developing human central nervous system. Neuron. (2016) 89:248–68. doi: 10.1016/j.neuron.2015.12.008, PMID: 26796689 PMC4959909

[10] Matejuk A Vandenbark AA Offner H. Cross-talk of the CNS with immune cells and functions in health and disease. Front Neurol. (2021) 12:672455. doi: 10.3389/fneur.2021.672455, PMID: 34135852 PMC8200536

[11] Sousa AMM Meyer KA Santpere G Gulden FO Sestan N. Evolution of the human nervous system function, structure, and development. Cell. (2017) 170:226–47. doi: 10.1016/j.cell.2017.06.036, PMID: 28708995 PMC5647789

[12] Wendimu MY Hooks SB. Microglia phenotypes in aging and neurodegenerative diseases. Cells. (2022) 11. doi: 10.3390/cells11132091, PMID: 35805174 PMC9266143

[13] Sanmarco LM Polonio CM Wheeler MA Quintana FJ. Functional immune cell-astrocyte interactions. J Exp Med. (2021) 218. doi: 10.1084/jem.20202715, PMID: 34292315 PMC8302447

[14] Kadry H Noorani B Cucullo L. A blood-brain barrier overview on structure, function, impairment, and biomarkers of integrity. Fluids Barriers CNS. (2020) 17:69. doi: 10.1186/s12987-020-00230-3, PMID: 33208141 PMC7672931

[15] Knox EG Aburto MR Clarke G Cryan JF O’Driscoll CM. The blood-brain barrier in aging and neurodegeneration. Mol Psychiatry. (2022) 27:2659–73. doi: 10.1038/s41380-022-01511-z, PMID: 35361905 PMC9156404

[16] Szlufik S Kopec K Szleszkowski S Koziorowski D. Glymphatic system pathology and neuroinflammation as two risk factors of neurodegeneration. Cells. (2024) 13. doi: 10.3390/cells13030286, PMID: 38334678 PMC10855155

[17] Iliff JJ Wang M Liao Y Plogg BA Peng W Gundersen GA. A paravascular pathway facilitates CSF flow through the brain parenchyma and the clearance of interstitial solutes, including amyloid beta. Sci Transl Med. (2012) 4:147ra11. doi: 10.1126/scitranslmed.3003748, PMID: 22896675 PMC3551275

[18] Sun B-L L-h W Yang T Sun J-Y Mao L-L Yang M-F. Lymphatic drainage system of the brain: A novel target for intervention of neurological diseases. Prog Neurobiol. (2018) 163–164:163–4 118–43. doi: 10.1016/j.pneurobio.2017.08.007, PMID: 28903061

[19] Colonna M Butovsky O. Microglia function in the central nervous system during health and neurodegeneration. Annu Rev Immunol. (2017) 35:441–68. doi: 10.1146/annurev-immunol-051116-052358, PMID: 28226226 PMC8167938

[20] Gao C Jiang J Tan Y Chen S. Microglia in neurodegenerative diseases: mechanism and potential therapeutic targets. Signal Transduct Target Ther. (2023) 8:359. doi: 10.1038/s41392-023-01588-0, PMID: 37735487 PMC10514343

[21] Tay TL Savage JC Hui CW Bisht K Tremblay ME. Microglia across the lifespan: from origin to function in brain development, plasticity and cognition. J Physiol. (2017) 595:1929–45. doi: 10.1113/JP272134, PMID: 27104646 PMC5350449

[22] Leite AOF Bento Torres Neto J Dos Reis RR Sobral LL de Souza ACP Trevia N. Unwanted exacerbation of the immune response in neurodegenerative disease: A time to review the impact. Front Cell Neurosci. (2021) 15:749595. doi: 10.3389/fncel.2021.749595, PMID: 34744633 PMC8570167

[23] Müller L Di Benedetto S. Aged brain and neuroimmune responses to COVID-19: post-acute sequelae and modulatory effects of behavioral and nutritional interventions. Immun Ageing. (2023) 20:17. doi: 10.1186/s12979-023-00341-z, PMID: 37046272 PMC10090758

[24] Norden DM Muccigrosso MM Godbout JP. Microglial priming and enhanced reactivity to secondary insult in aging, and traumatic CNS injury, and neurodegenerative disease. Neuropharmacology. (2015) 96:29–41. doi: 10.1016/j.neuropharm.2014.10.028, PMID: 25445485 PMC4430467

[25] Rao JS Kellom M Kim HW Rapoport SI Reese EA. Neuroinflammation and synaptic loss. Neurochem Res. (2012) 37:903–10. doi: 10.1007/s11064-012-0708-2, PMID: 22311128 PMC3478877

[26] Fang L-P Bai X. Oligodendrocyte precursor cells: the multitaskers in the brain. Pflügers Archiv - Eur J Physiol. (2023) 475:1035–44. doi: 10.1007/s00424-023-02837-5, PMID: 37401986 PMC10409806

[27] Benarroch E. What are the roles of oligodendrocyte precursor cells in normal and pathologic conditions? Neurology. (2023) 101:958–65. doi: 10.1212/WNL.0000000000208000, PMID: 37985182 PMC10663025

[28] Kirby L Jin J Cardona JG Smith MD Martin KA Wang J. Oligodendrocyte precursor cells present antigen and are cytotoxic targets in inflammatory demyelination. Nat Commun. (2019) 10:3887. doi: 10.1038/s41467-019-11638-3, PMID: 31467299 PMC6715717

[29] Guarnieri G Sarchielli E Comeglio P Herrera-Puerta E Piaceri I Nacmias B. Tumor necrosis factor alpha influences phenotypic plasticity and promotes epigenetic changes in human basal forebrain cholinergic neuroblasts. Int J Mol Sci. (2020) 21. doi: 10.3390/ijms21176128, PMID: 32854421 PMC7504606

[30] Kuno R Yoshida Y Nitta A Nabeshima T Wang J Sonobe Y. The role of TNF-alpha and its receptors in the production of NGF and GDNF by astrocytes. Brain Res. (2006) 1116:12–8. doi: 10.1016/j.brainres.2006.07.120, PMID: 16956589

[31] Alboni S Maggi L. Editorial: cytokines as players of neuronal plasticity and sensitivity to environment in healthy and pathological brain. Front Cell Neurosci. (2015) 9:508. doi: 10.3389/fncel.2015.00508, PMID: 26793060 PMC4709412

[32] Lim SH Park E You B Jung Y Park AR Park SG. Neuronal synapse formation induced by microglia and interleukin 10. PloS One. (2013) 8:e81218. doi: 10.1371/journal.pone.0081218, PMID: 24278397 PMC3838367

[33] Stellwagen D Malenka RC. Synaptic scaling mediated by glial TNF-alpha. Nature. (2006) 440:1054–9. doi: 10.1038/nature04671, PMID: 16547515

[34] Soteros BM Sia GM. Complement and microglia dependent synapse elimination in brain development. WIREs Mech Dis. (2022) 14:e1545. doi: 10.1002/wsbm.1545, PMID: 34738335 PMC9066608

[35] Wheeler MA Quintana FJ. The neuroimmune connectome in health and disease. Nature. (2025) 638:333–42. doi: 10.1038/s41586-024-08474-x, PMID: 39939792 PMC12039074

[36] Zhao S Umpierre AD Wu LJ. Tuning neural circuits and behaviors by microglia in the adult brain. Trends Neurosci. (2024) 47:181–94. doi: 10.1016/j.tins.2023.12.003, PMID: 38245380 PMC10939815

[37] Müller L Di Benedetto S. Neuroimmune crosstalk in chronic neuroinflammation: microglial interactions and immune modulation. Front Cell Neurosci. (2025) 19:1575022. doi: 10.3389/fncel.2025.1575022, PMID: 40260075 PMC12009833

[38] Bianchi ME. DAMPs, PAMPs and alarmins: all we need to know about danger. J Leukoc Biol. (2007) 81:1–5. doi: 10.1189/jlb.0306164, PMID: 17032697

[39] Venereau E De Leo F Mezzapelle R Careccia G Musco G Bianchi ME. HMGB1 as biomarker and drug target. Pharmacol Res. (2016) 111:534–44. doi: 10.1016/j.phrs.2016.06.031, PMID: 27378565

[40] Hanisch U-K Kettenmann H. Microglia: active sensor and versatile effector cells in the normal and pathologic brain. Nat Neurosci. (2007) 10:1387–94. doi: 10.1038/nn1997, PMID: 17965659

[41] Labzin LI Heneka MT Latz E. Innate immunity and neurodegeneration. Annu Rev Med. (2018) 69:437–49. doi: 10.1146/annurev-med-050715-104343, PMID: 29106805

[42] Heneka MT Carson MJ El Khoury J Landreth GE Brosseron F Feinstein DL. Neuroinflammation in alzheimer’s disease. Lancet Neurol. (2015) 14:388–405. doi: 10.1016/S1474-4422(15)70016-5 25792098 PMC5909703

[43] Heneka MT Kummer MP Latz E. Innate immune activation in neurodegenerative disease. Nat Rev Immunol. (2014) 14:463–77. doi: 10.1038/nri3705, PMID: 24962261

[44] Perea JR Bolos M Avila J. Microglia in alzheimer’s disease in the context of tau pathology. Biomolecules. (2020) 10. doi: 10.3390/biom10101439, PMID: 33066368 PMC7602223

[45] Kim C Ho DH Suk JE You S Michael S Kang J. Neuron-released oligomeric alpha-synuclein is an endogenous agonist of TLR2 for paracrine activation of microglia. Nat Commun. (2013) 4:1562. doi: 10.1038/ncomms2534, PMID: 23463005 PMC4089961

[46] Müller L Di Benedetto S. Aging brain: exploring the interplay between bone marrow aging, immunosenescence, and neuroinflammation. Front Immunol. (2024) 15. doi: 10.3389/fimmu.2024.1393324, PMID: 38638424 PMC11024322

[47] Franceschi C Campisi J. Chronic inflammation (inflammaging) and its potential contribution to age-associated diseases. J Gerontol A Biol Sci Med Sci. (2014) 69 Suppl 1:S4–9. doi: 10.1093/gerona/glu057, PMID: 24833586

[48] Holbrook JA Jarosz-Griffiths HH Caseley E Lara-Reyna S Poulter JA Williams-Gray CH. Neurodegenerative disease and the NLRP3 inflammasome. Front Pharmacol. (2021) 12:643254. doi: 10.3389/fphar.2021.643254, PMID: 33776778 PMC7987926

[49] Boahen A Hu D Adams MJ Nicholls PK Greene WK Ma B. Bidirectional crosstalk between the peripheral nervous system and lymphoid tissues/organs. Front Immunol. (2023) 14:1254054. doi: 10.3389/fimmu.2023.1254054, PMID: 37767094 PMC10520967

[50] Kawai T Akira S. The role of pattern-recognition receptors in innate immunity: update on Toll-like receptors. Nat Immunol. (2010) 11:373–84. doi: 10.1038/ni.1863, PMID: 20404851

[51] Ransohoff RM. How neuroinflammation contributes to neurodegeneration. Science. (2016) 353:777–83. doi: 10.1126/science.aag2590, PMID: 27540165

[52] Block ML Calderón-Garcidueñas L. Air pollution: mechanisms of neuroinflammation and CNS disease. Trends Neurosciences. (2009) 32:506–16. doi: 10.1016/j.tins.2009.05.009, PMID: 19716187 PMC2743793

[53] Thomson EM. Air pollution, stress, and allostatic load: linking systemic and central nervous system impacts. J Alzheimers Dis. (2019) 69:597–614. doi: 10.3233/JAD-190015, PMID: 31127781 PMC6598002

[54] Loane DJ Kumar A. Microglia in the TBI brain: The good, the bad, and the dysregulated. Exp Neurol. (2016) 275 Pt 3:316–27. doi: 10.1016/j.expneurol.2015.08.018, PMID: 26342753 PMC4689601

[55] Ulrich JD Holtzman DM. TREM2 function in alzheimer’s disease and neurodegeneration. ACS Chem Neurosci. (2016) 7:420–7. doi: 10.1021/acschemneuro.5b00313, PMID: 26854967

[56] Colonna M Wang Y. TREM2 variants: new keys to decipher Alzheimer disease pathogenesis. Nat Rev Neurosci. (2016) 17:201–7. doi: 10.1038/nrn.2016.7, PMID: 26911435

[57] Shi Y Yamada K Liddelow SA Smith ST Zhao L Luo W. ApoE4 markedly exacerbates tau-mediated neurodegeneration in a mouse model of tauopathy. Nature. (2017) 549:523–7. doi: 10.1038/nature24016, PMID: 28959956 PMC5641217

[58] Tai LM Ghura S Koster KP Liakaite V Maienschein-Cline M Kanabar P. APOE-modulated Abeta-induced neuroinflammation in Alzheimer’s disease: current landscape, novel data, and future perspective. J Neurochem. (2015) 133:465–88. doi: 10.1111/jnc.13072, PMID: 25689586 PMC4400246

[59] Liu C-C Kanekiyo T Xu H Bu G. Apolipoprotein E and Alzheimer disease: risk, mechanisms and therapy. Nat Rev Neurology. (2013) 9:106–18. doi: 10.1038/nrneurol.2012.263, PMID: 23296339 PMC3726719

[60] Jain M Singh MK Shyam H Mishra A Kumar S Kumar A. Role of JAK/STAT in the neuroinflammation and its association with neurological disorders. Ann Neurosci. (2021) 28:191–200. doi: 10.1177/09727531211070532, PMID: 35341232 PMC8948319

[61] Kempuraj D Thangavel R Natteru PA Selvakumar GP Saeed D Zahoor H. Neuroinflammation induces neurodegeneration. J Neurol Neurosurg Spine. (2016) 1. PMC5260818 28127589

[62] Ransohoff RM. A polarizing question: do M1 and M2 microglia exist? Nat Neurosci. (2016) 19:987–91. doi: 10.1038/nn.4338, PMID: 27459405

[63] Colombo G Cubero RJA Kanari L Venturino A Schulz R Scolamiero M. A tool for mapping microglial morphology, morphOMICs, reveals brain-region and sex-dependent phenotypes. Nat Neurosci. (2022) 25:1379–93. doi: 10.1038/s41593-022-01167-6, PMID: 36180790 PMC9534764

[64] Paolicelli RC Sierra A Stevens B Tremblay ME Aguzzi A Ajami B. Microglia states and nomenclature: A field at its crossroads. Neuron. (2022) 110:3458–83. doi: 10.1016/j.neuron.2022.10.020, PMID: 36327895 PMC9999291

[65] Chen LX Zhang MD Xu HF Ye HQ Chen DF Wang PS. Single-nucleus RNA sequencing reveals the spatiotemporal dynamics of disease-associated microglia in amyotrophic lateral sclerosis. Res (Wash D C). (2024) 7:0548. doi: 10.34133/research.0548, PMID: 39664295 PMC11632836

[66] Hou J Chen Y Grajales-Reyes G Colonna M. TREM2 dependent and independent functions of microglia in Alzheimer’s disease. Mol Neurodegener. (2022) 17:84. doi: 10.1186/s13024-022-00588-y, PMID: 36564824 PMC9783481

[67] Gratuze M Schlachetzki JCM D’Oliveira Albanus R Jain N Novotny B Brase L. TREM2-independent microgliosis promotes tau-mediated neurodegeneration in the presence of ApoE4. Neuron. (2023) 111:202–19. e7. doi: 10.1016/j.neuron.2022.10.022, PMID: 36368315 PMC9852006

[68] Haney MS Palovics R Munson CN Long C Johansson PK Yip O. APOE4/4 is linked to damaging lipid droplets in Alzheimer’s disease microglia. Nature. (2024) 628:154–61. doi: 10.1038/s41586-024-07185-7, PMID: 38480892 PMC10990924

[69] Masuda T Sankowski R Staszewski O Bottcher C Sagar AL. Spatial and temporal heterogeneity of mouse and human microglia at single-cell resolution. Nature. (2019) 566:388–92. doi: 10.1038/s41586-019-0924-x, PMID: 30760929

[70] Keren-Shaul H Spinrad A Weiner A Matcovitch-Natan O Dvir-Szternfeld R Ulland TK. A unique microglia type associated with restricting development of alzheimer’s disease. Cell. (2017) 169:1276–90 e17. doi: 10.1016/j.cell.2017.05.018, PMID: 28602351

[71] Deczkowska A Weiner A Amit I. The physiology, pathology, and potential therapeutic applications of the TREM2 signaling pathway. Cell. (2020) 181:1207–17. doi: 10.1016/j.cell.2020.05.003, PMID: 32531244

[72] Rangaraju S Dammer EB Raza SA Rathakrishnan P Xiao H Gao T. Identification and therapeutic modulation of a pro-inflammatory subset of disease-associated-microglia in Alzheimer’s disease. Mol Neurodegener. (2018) 13:24. doi: 10.1186/s13024-018-0254-8, PMID: 29784049 PMC5963076

[73] Ji K Tsirka SE. Inflammation modulates expression of laminin in the central nervous system following ischemic injury. J Neuroinflammation. (2012) 9:159. doi: 10.1186/1742-2094-9-159, PMID: 22759265 PMC3414761

[74] Logsdon AF Rhea EM Reed M Banks WA Erickson MA. The neurovascular extracellular matrix in health and disease. Exp Biol Med (Maywood). (2021) 246:835–44. doi: 10.1177/1535370220977195, PMID: 33302738 PMC8719034

[75] Takano T Tian GF Peng W Lou N Libionka W Han X. Astrocyte-mediated control of cerebral blood flow. Nat Neurosci. (2006) 9:260–7. doi: 10.1038/nn1623, PMID: 16388306

[76] Kunchok A Zekeridou A McKeon A. Autoimmune glial fibrillary acidic protein astrocytopathy. Curr Opin Neurol. (2019) 32:452–8. doi: 10.1097/WCO.0000000000000676, PMID: 30724768 PMC6522205

[77] Hol EM Pekny M. Glial fibrillary acidic protein (GFAP) and the astrocyte intermediate filament system in diseases of the central nervous system. Curr Opin Cell Biol. (2015) 32:121–30. doi: 10.1016/j.ceb.2015.02.004, PMID: 25726916

[78] Patani R Hardingham GE Liddelow SA. Functional roles of reactive astrocytes in neuroinflammation and neurodegeneration. Nat Rev Neurol. (2023) 19:395–409. doi: 10.1038/s41582-023-00822-1, PMID: 37308616

[79] Sofroniew MV. Molecular dissection of reactive astrogliosis and glial scar formation. Trends Neurosci. (2009) 32:638–47. doi: 10.1016/j.tins.2009.08.002, PMID: 19782411 PMC2787735

[80] Liu T Zhang L Joo D Sun SC. NF-kappaB signaling in inflammation. Signal Transduct Target Ther. (2017) 2:17023–. doi: 10.1038/sigtrans.2017.23, PMID: 29158945 PMC5661633

[81] Prevot V. Glial control of neuronal function. Nat Rev Endocrinol. (2022) 18:195. doi: 10.1038/s41574-022-00640-3, PMID: 35115720

[82] Burda JE Sofroniew MV. Reactive gliosis and the multicellular response to CNS damage and disease. Neuron. (2014) 81:229–48. doi: 10.1016/j.neuron.2013.12.034, PMID: 24462092 PMC3984950

[83] Kim EK Choi EJ. Pathological roles of MAPK signaling pathways in human diseases. Biochim Biophys Acta. (2010) 1802:396–405. doi: 10.1016/j.bbadis.2009.12.009, PMID: 20079433

[84] Hopfner KP Hornung V. Molecular mechanisms and cellular functions of cGAS-STING signaling. Nat Rev Mol Cell Biol. (2020) 21:501–21. doi: 10.1038/s41580-020-0244-x, PMID: 32424334

[85] Paul BD Snyder SH Bohr VA. Signaling by cGAS-STING in neurodegeneration, neuroinflammation, and aging. Trends Neurosci. (2021) 44:83–96. doi: 10.1016/j.tins.2020.10.008, PMID: 33187730 PMC8662531

[86] Zhang W Chen Y Pei H. C1q and central nervous system disorders. Front Immunol. (2023) 14:1145649. doi: 10.3389/fimmu.2023.1145649, PMID: 37033981 PMC10076750

[87] Liddelow SA Guttenplan KA Clarke LE Bennett FC Bohlen CJ Schirmer L. Neurotoxic reactive astrocytes are induced by activated microglia. Nature. (2017) 541:481–7. doi: 10.1038/nature21029, PMID: 28099414 PMC5404890

[88] Norden DM Fenn AM Dugan A Godbout JP. TGFbeta produced by IL-10 redirected astrocytes attenuates microglial activation. Glia. (2014) 62:881–95. doi: 10.1002/glia.22647, PMID: 24616125 PMC4061706

[89] Yang R Yang B Liu W Tan C Chen H Wang X. Emerging role of non-coding RNAs in neuroinflammation mediated by microglia and astrocytes. J Neuroinflammation. (2023) 20:173. doi: 10.1186/s12974-023-02856-0, PMID: 37481642 PMC10363317

[90] Pamies D Sartori C Schvartz D Gonzalez-Ruiz V Pellerin L Nunes C. Neuroinflammatory response to TNFalpha and IL1beta cytokines is accompanied by an increase in glycolysis in human astrocytes *in vitro* . . Int J Mol Sci. (2021) 22. doi: 10.3390/ijms22084065, PMID: 33920048 PMC8071021

[91] Jurgens HA Johnson RW. Dysregulated neuronal-microglial cross-talk during aging, stress and inflammation. Exp Neurol. (2012) 233:40–8. doi: 10.1016/j.expneurol.2010.11.014, PMID: 21110971 PMC3071456

[92] Chavda V Singh K Patel V Mishra M Mishra AK. Neuronal glial crosstalk: specific and shared mechanisms in alzheimer’s disease. Brain Sci. (2022) 12. doi: 10.3390/brainsci12010075, PMID: 35053818 PMC8773743

[93] Badimon A Strasburger HJ Ayata P Chen X Nair A Ikegami A. Negative feedback control of neuronal activity by microglia. Nature. (2020) 586:417–23. doi: 10.1038/s41586-020-2777-8, PMID: 32999463 PMC7577179

[94] Duarte Azevedo M Sander S Tenenbaum L. GDNF. A neuron-derived factor upregulated in glial cells during disease. J Clin Med. (2020) 9. doi: 10.3390/jcm9020456, PMID: 32046031 PMC7073520

[95] Dowlati Y Herrmann N Swardfager W Liu H Sham L Reim EK. A meta-analysis of cytokines in major depression. Biol Psychiatry. (2010) 67:446–57. doi: 10.1016/j.biopsych.2009.09.033, PMID: 20015486

[96] Solmi M Suresh Sharma M Osimo EF Fornaro M Bortolato B Croatto G. Peripheral levels of C-reactive protein, tumor necrosis factor-alpha, interleukin-6, and interleukin-1beta across the mood spectrum in bipolar disorder: A meta-analysis of mean differences and variability. Brain Behav Immun. (2021) 97:193–203. doi: 10.1016/j.bbi.2021.07.014, PMID: 34332041

[97] Kim H Baek S-H Kim J-W Ryu S Lee J-Y Kim J-M. Inflammatory markers of symptomatic remission at 6 months in patients with first-episode schizophrenia. Schizophrenia. (2023) 9:68. doi: 10.1038/s41537-023-00398-1, PMID: 37794014 PMC10550944

[98] Elgellaie A Thomas SJ Kaelle J Bartschi J Larkin T. Pro-inflammatory cytokines IL-1alpha, IL-6 and TNF-alpha in major depressive disorder: Sex-specific associations with psychological symptoms. Eur J Neurosci. (2023) 57:1913–28. doi: 10.1111/ejn.15992, PMID: 37070163

[99] Min X Wang G Cui Y Meng P Hu X Liu S. Association between inflammatory cytokines and symptoms of major depressive disorder in adults. Front Immunol. (2023) 14:1110775. doi: 10.3389/fimmu.2023.1110775, PMID: 36860860 PMC9968963

[100] Jadhav KK Daouk J Kurkinen K Kraav SL Eriksson P Tolmunen T. Blood cytokines in major depressive disorder in drug-naive adolescents: A systematic review and meta-analysis. J Affect Disord. (2025) 372:48–55. doi: 10.1016/j.jad.2024.11.071, PMID: 39603515

[101] Ebrahimi R Shahrokhi Nejad S Falah Tafti M Karimi Z Sadr SR Ramadhan Hussein D. Microglial activation as a hallmark of neuroinflammation in Alzheimer’s disease. Metab Brain Dis. (2025) 40:207. doi: 10.1007/s11011-025-01631-9, PMID: 40381069

[102] Kwon HS Koh S-H. Neuroinflammation in neurodegenerative disorders: the roles of microglia and astrocytes. Trans Neurodegeneration. (2020) 9:42. doi: 10.1186/s40035-020-00221-2, PMID: 33239064 PMC7689983

[103] Meraz-Rios MA Toral-Rios D Franco-Bocanegra D Villeda-Hernandez J Campos-Pena V. Inflammatory process in alzheimer’s disease. Front Integr Neurosci. (2013) 7:59. doi: 10.3389/fnint.2013.00059, PMID: 23964211 PMC3741576

[104] Parhizkar S Holtzman DM. APOE mediated neuroinflammation and neurodegeneration in Alzheimer’s disease. Semin Immunol. (2022) 59:101594. doi: 10.1016/j.smim.2022.101594, PMID: 35232622 PMC9411266

[105] Wijesinghe P Li HR Ai Z Campbell M Chen SX Xi J. Apolipoprotein E dysfunction in Alzheimer’s disease: a study on miRNA regulation, glial markers, and amyloid pathology. Front Aging Neurosci. (2024) 16:1495615. doi: 10.3389/fnagi.2024.1495615, PMID: 39744521 PMC11688329

[106] Condello C Yuan P Grutzendler J. Microglia-mediated neuroprotection, TREM2, and alzheimer’s disease: evidence from optical imaging. Biol Psychiatry. (2018) 83:377–87. doi: 10.1016/j.biopsych.2017.10.007, PMID: 29169609 PMC5767550

[107] Hirsch EC Hunot S. Neuroinflammation in Parkinson’s disease: a target for neuroprotection? Lancet Neurol. (2009) 8:382–97. doi: 10.1016/S1474-4422(09)70062-6 19296921

[108] Perry VH. Innate inflammation in Parkinson’s disease. Cold Spring Harb Perspect Med. (2012) 2:a009373. doi: 10.1101/cshperspect.a009373, PMID: 22951445 PMC3426823

[109] Gordon R Albornoz EA Christie DC Langley MR Kumar V Mantovani S. Inflammasome inhibition prevents alpha-synuclein pathology and dopaminergic neurodegeneration in mice. Sci Transl Med. (2018) 10. doi: 10.1126/scitranslmed.aah4066, PMID: 30381407 PMC6483075

[110] Tansey MG Romero-Ramos M. Immune system responses in Parkinson’s disease: Early and dynamic. Eur J Neurosci. (2019) 49:364–83. doi: 10.1111/ejn.14290, PMID: 30474172 PMC6391192

[111] Weiss F Hughes L Fu Y Bardy C Halliday GM Dzamko N. Astrocytes contribute to toll-like receptor 2-mediated neurodegeneration and alpha-synuclein pathology in a human midbrain Parkinson’s model. Transl Neurodegener. (2024) 13:62. doi: 10.1186/s40035-024-00448-3, PMID: 39681872 PMC11648304

[112] Cabezudo D Tsafaras G Van Acker E Van den Haute C Baekelandt V. Mutant LRRK2 exacerbates immune response and neurodegeneration in a chronic model of experimental colitis. Acta Neuropathol. (2023) 146:245–61. doi: 10.1007/s00401-023-02595-9, PMID: 37289222 PMC10328902

[113] Lind-Holm Mogensen F Sousa C Ameli C Badanjak K Pereira SL Muller A. PARK7/DJ-1 deficiency impairs microglial activation in response to LPS-induced inflammation. J Neuroinflammation. (2024) 21:174. doi: 10.1186/s12974-024-03164-x, PMID: 39014482 PMC11253405

[114] Zhang M Li C Ren J Wang H Yi F Wu J. The double-faceted role of leucine-rich repeat kinase 2 in the immunopathogenesis of parkinson’s disease. Front Aging Neurosci. (2022) 14:909303. doi: 10.3389/fnagi.2022.909303, PMID: 35645775 PMC9131027

[115] Charabati M Wheeler MA Weiner HL Quintana FJ. Multiple sclerosis: Neuroimmune crosstalk and therapeutic targeting. Cell. (2023) 186:1309–27. doi: 10.1016/j.cell.2023.03.008, PMID: 37001498 PMC10119687

[116] Xu X Han Y Zhang B Ren Q Ma J Liu S. Understanding immune microenvironment alterations in the brain to improve the diagnosis and treatment of diverse brain diseases. Cell Communication Signaling. (2024) 22:132. doi: 10.1186/s12964-024-01509-w, PMID: 38368403 PMC10874090

[117] Kooistra SM Schirmer L. Multiple sclerosis: glial cell diversity in time and space. Glia. (2025) 73:574–90. doi: 10.1002/glia.24655, PMID: 39719685 PMC11784844

[118] Ludwin SK Rao V Moore CS Antel JP. Astrocytes in multiple sclerosis. Mult Scler. (2016) 22:1114–24. doi: 10.1177/1352458516643396, PMID: 27207458

[119] Rossi B Santos-Lima B Terrabuio E Zenaro E Constantin G. Common peripheral immunity mechanisms in multiple sclerosis and alzheimer’s disease. Front Immunol. (2021) 12:639369. doi: 10.3389/fimmu.2021.639369, PMID: 33679799 PMC7933037

[120] Shi Y Wei B Li L Wang B Sun M. Th17 cells and inflammation in neurological disorders: Possible mechanisms of action. Front Immunol. (2022) 13:932152. doi: 10.3389/fimmu.2022.932152, PMID: 35935951 PMC9353135

[121] Hu H Li H Li R Liu P Liu H. Re-establishing immune tolerance in multiple sclerosis: focusing on novel mechanisms of mesenchymal stem cell regulation of Th17/Treg balance. J Transl Med. (2024) 22:663. doi: 10.1186/s12967-024-05450-x, PMID: 39010157 PMC11251255

[122] Cree BAC Berger JR Greenberg B. The evolution of anti-CD20 treatment for multiple sclerosis: optimization of antibody characteristics and function. CNS Drugs. (2025) 39:545–64. doi: 10.1007/s40263-025-01182-8, PMID: 40180777 PMC12058931

[123] Galota F Marcheselli S De Biasi S Gibellini L Vitetta F Fiore A. Impact of high-efficacy therapies for multiple sclerosis on B cells. Cells. (2025) 14. doi: 10.3390/cells14080606, PMID: 40277931 PMC12025603

[124] International Multiple Sclerosis Genetics C, Wellcome Trust Case Control C Sawcer S Hellenthal G Pirinen M Spencer CC. Genetic risk and a primary role for cell-mediated immune mechanisms in multiple sclerosis. Nature. (2011) 476:214–9. doi: 10.1038/nature10251, PMID: 21833088 PMC3182531

[125] International Multiple Sclerosis Genetics C Hafler DA Compston A Sawcer S Lander ES Daly MJ. Risk alleles for multiple sclerosis identified by a genomewide study. N Engl J Med. (2007) 357:851–62. doi: 10.1056/NEJMoa073493, PMID: 17660530

[126] Maier LM Lowe CE Cooper J Downes K Anderson DE Severson C. IL2RA genetic heterogeneity in multiple sclerosis and type 1 diabetes susceptibility and soluble interleukin-2 receptor production. PloS Genet. (2009) 5:e1000322. doi: 10.1371/journal.pgen.1000322, PMID: 19119414 PMC2602853

